# The effect of Animal-assisted therapy on prosocial behavior and emotional regulation in autistic children with varying verbal abilities: A pilot study

**DOI:** 10.1371/journal.pone.0326085

**Published:** 2025-07-01

**Authors:** Michele Kilmer, Minju Hong, Victor Akakpo, Terria Hawley, Danielle Randolph, Sarah Huetter, Allison Reichel, Madelyn Bowden

**Affiliations:** 1 Eleanor Mann School of Nursing, The University of Arkansas, Fayetteville, Arkansas, United States of America; 2 College of Social Science, Chung-Ang University, Dongjak-gu, Seoul, Republic of Korea; Father Muller Charitable Institutions, INDIA

## Abstract

**Background:**

The use of animal-assisted therapy (AAT) has increased in the pediatric autism population. However, studies detailing differences in human-animal interaction between autistic children and animals along with the longevity of reported outcomes associated with AAT need further exploration. The purpose of this pilot study was to evaluate these factors.

**Methods:**

A quantitative research design with convenience sampling was used to categorize pediatric participants into two groups (nonverbal or verbal) based on their verbality. Two human-animal ethogram and two questionnaires were utilized to assess behavior during and apart from AAT sessions. A total of 2,281 interactions and behaviors occurring during AAT sessions were examined.

**Results:**

Both groups interacted well with the canine. The verbal group interacted mostly with commands while the nonverbal group showed more affectionate behaviors.

**Conclusion:**

Mental health practitioners can use canines to enhance therapeutic outcomes in autistic children regardless of the child’s verbality.

## Introduction

Autism spectrum disorder (ASD) is a neurodevelopmental condition occurring in all racial, ethnic, and socioeconomic groups. It is associated with deficits in social interaction and repetitive interests/behaviors [1], increased morbidity and mortality [2], and deficits in speech-language, fine, and gross motor development [3]. Many autistic children display immature emotional regulation, defined as the ability to monitor, evaluate, and change one’s emotional state to achieve a goal [1,4,5]. However, autistic children with greater receptive language abilities may demonstrate a more mature emotional regulation response [4], indicating a correlation between language ability and emotional regulation. Nonetheless, autistic children with average or above average cognitive and verbal abilities may also demonstrate deficits in functional and social communication [6]. Additionally, children with ASD may exhibit higher levels of involuntary emotional dysregulation behaviors, such as rumination and intrusive thoughts [7]. These behaviors underscore the importance of personalized interventions and support strategies that can enhance social and emotional skills in autistic children while considering their unique verbal abilities and needs.

As the prevalence of ASD continues to increase [8], early intervention to address developmental delays, social deficits, and emotional regulation is recommended [2]. An increase in animal-assisted therapy (AAT) to address these skills is noted in the pediatric ASD population [9]. Animal-assisted therapy supports established therapy goals through direct and purposeful interaction with animals [10]. The formal utilization of animals in healthcare specifically within therapy dates to the mid-to-late 20th century when Boris M. Levinson used canines to comfort children during therapy sessions [11–13]. Furthermore, Lev Vygotsky’s developmental theory supports interaction with an outside source, such as peers and adults, to develop self-regulation in children [14]. However, canines can be used as the outside source with which the child can learn emotional regulation [15]. Pediatric therapists have used AAT to promote verbal and nonverbal communication [14], increase prosocial behaviors [16,17], and promote emotional regulation in children with ASD both during and apart from sessions [17,18]. Positive verbal and nonverbal social behaviors and reduced isolating and aggressive behaviors have been noted during AAT sessions in children with ASD [19]. Moreover, AAT resulted in a statistical improvement in both communication and social interaction skills in a small sample of preschool-aged children diagnosed with ASD [20].

Canines show notable potential in providing unique treatment modality for autistic children with limited verbal abilities. The presence of a canine in therapy sessions specifically increased both the frequency and duration of positive social behaviors [16]. It also led to enhanced emotional regulation and attention, resulting in a calming effect during the session [18]. One possible explanation for these results is that children with ASD may perceive greater social reward from animal faces compared to human faces, which could indicate enhanced emotional arousal and regulation during AAT sessions [21]. Canines can be used to engage children in simple social activities that do not require verbal communication, such as playing fetch or walking the canine on a leash [16]. Furthermore, autistic children with speech delay may understand canine nonverbal communication more than human verbal communication, which involves complex verbal and social cues that can be difficult to decode [22–24].

Canines trained in ASD tasks are the third most requested service animal in the United States due to their success in promoting socialization and emotional regulation. However, many families cannot afford to purchase or home a service canine [13]. Few, if any, studies have examined if a canine trained to serve multiple children could be as effective as a service canine trained for one child. Moreover, despite the increasing popularity of AAT in the therapeutic plan for autistic children, the usage of animals, specifically canines, with patients of varying verbal abilities has yet to be formally analyzed. For these reasons, this present study aims to investigate interactions within AAT sessions between a trained canine and children with ASD of varying verbal abilities to lay a foundational understanding of human-canine bonding. Furthermore, this study will examine if behavioral changes occur apart from AAT sessions, as seen in children with ASD who have service canines, to note if AAT could produce similar effects for families who cannot afford a service canine. This new information can guide mental health practitioners seeking to include canines in their clinical practice.

## Materials and methods

### Aim

This mixed-methods study aims to investigate interactions within AAT sessions between a trained canine and children with ASD of varying verbal abilities to lay a foundational understanding of best practice for AAT use. Evaluating prosocial behavior and emotional regulation occurring during AAT sessions between autistic children with varying verbality can ascertain if autistic children respond differently to animals based on their verbal abilities. Furthermore, investigating changes in prosocial behavior and emotional regulation apart from AAT sessions provides new insight into the longevity of outcomes associated with AAT. This new information can guide mental health practitioners seeking to include canines in their clinical practice to enhance therapy sessions and meet therapeutic outcomes. The following research questions were established to attain these study objectives:

1 Do interactions between a trained therapy canine and autistic children differ depending on the child’s verbal abilities?
2 Is there a difference in prosocial behavior and emotional regulation in animal-assisted therapy sessions among children with varying verbal abilities?
3 Is there a difference in prosocial behavior and emotional regulation apart from animal-assisted therapy sessions among children with varying verbal abilities?

### Ethics

This study was approved by the internal review board at the university using protocol number 2201382953 and the institutional animal care and use committee prior to implementation. The clinical trial registration number is NCT06687850. Written informed consent was obtained at the start of the first session on all caregivers and pediatric participants ages 8 years and older who were cognitively able to give consent.

### Design

This present study used standardized instruments to investigate human-animal intervention (HAI) occurring during AAT sessions and prosocial behavior and emotional regulation demonstrated by participants outside of AAT sessions. SPSS and Microsoft Excel were used to run the analysis. A total of 144 sessions were conducted, 84 of which were recorded and coded to analyze HAI during sessions. Independent t-tests were used to analyze the differences in human-animal interaction among children with ASD with varying verbal abilities from the recorded sessions. The homogeneity of the variance (HOV) assumption between two groups was checked using Levene’s test for equality of variances in SPSS program before conducting the t-tests analysis. Results of t-tests with adjusted degrees of freedom value were reported if the HOV was violated. The null hypothesis was set to reflect no significant difference in HAI between verbal and nonverbal groups for the independent t-tests; therefore, a significant difference in the human-animal interaction behaviors regarding the children’s verbal abilities could be confirmed if the t-test results reject the null hypothesis. Cohen’s *d* was used to report the effect sizes of the independent t-tests since Cohen’s d is one of the most commonly reported effect sizes in group comparison analysis. Values up to 0.20 were interpreted as small, 0.50 as medium, and 0.80 as large effect sizes [25]. Due to the smaller sample size, descriptive statistics were utilized to compare prosocial behavior and emotional regulation occurring apart from AAT sessions between the two groups based on the self-report questionnaires completed by caregivers. This study was not registered as a clinical trial before enrollment; however, the authors confirm that ongoing trials for animal-assisted therapy will be registered.

### Sample

**Fig 1:**
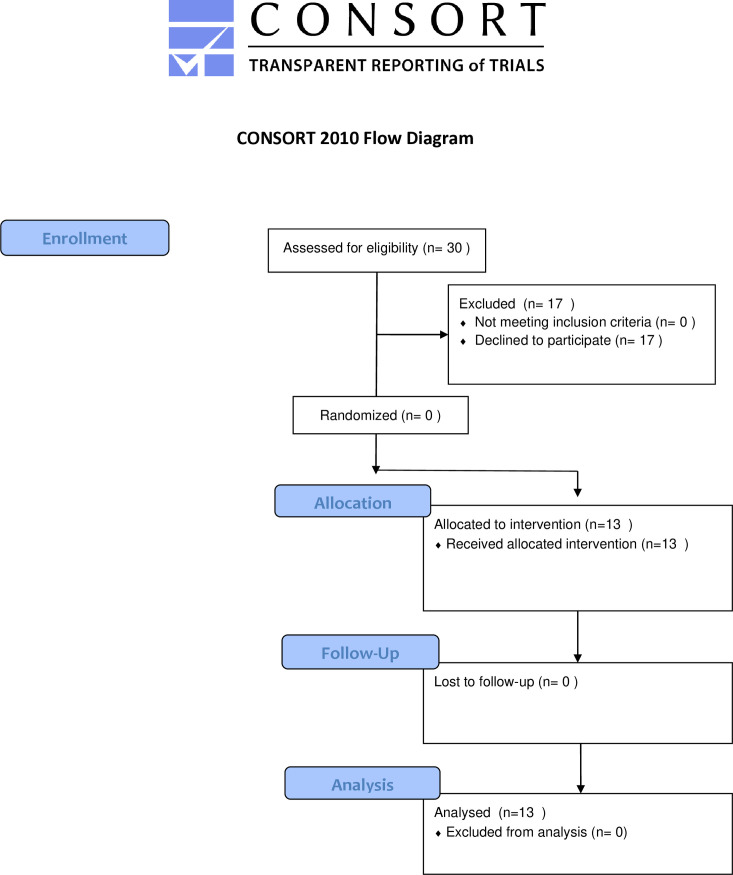
CONSORT flow diagram. (A) This diagram illustrates the flow of participants through each phase of the study, in accordance with CONSORT guidelines. It includes the number of participants assessed for eligibility, those who were excluded because they chose not to participate in the study, those who received animal-assisted therapy, those who were lost to follow-up, and those included in the final analysis.

Convenience sampling was used to recruit pediatric participants, ages 2 to 21 years, identified at risk for ASD by community healthcare providers and referred to an ASD program supported by the university for a diagnostic evaluation. The university has a clinic on campus where the AAT sessions were held. Eligible participants included children referred to the program for an ASD diagnostic evaluation and were not allergic to or afraid of canines. Caregivers were informed of the AAT study during the telehealth follow-up evaluation which occurred after the diagnostic appointment. See Fig 1 for the CONSORT Flow Diagram.

**Fig 2:**
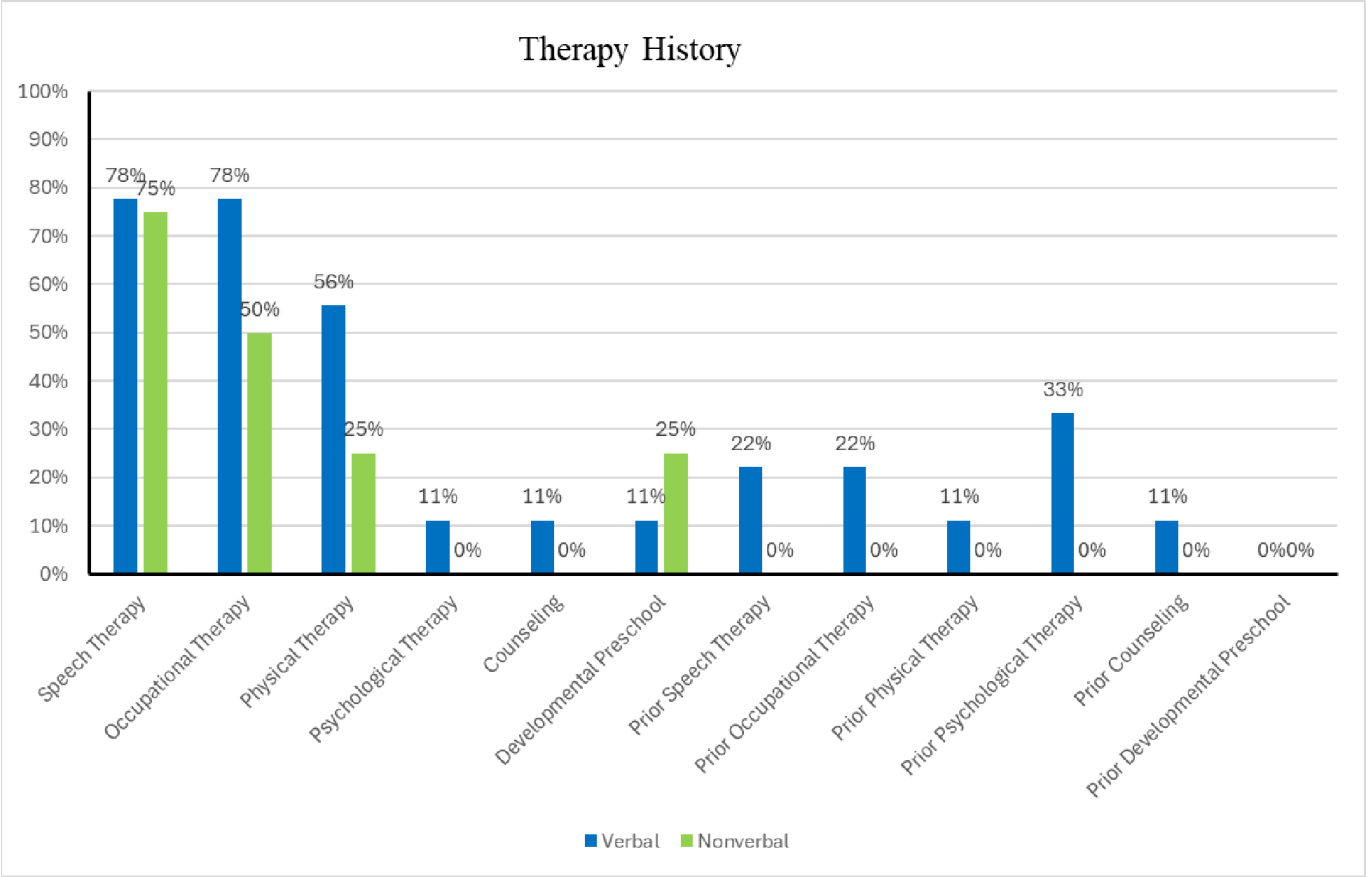
Percentages of therapy history. (A) Distribution of participants based on their history of prior therapy. Percentages represent the proportion of participants in both groups who reported receiving specific types of therapy prior to or during the study period.

A total of 13 children, 12 males and one female, enrolled in the AAT study from June 2022 to August 2023. Seventy-seven percent (n = 10) were diagnosed with ASD, 15% (n = 2) were diagnosed with developmental delay and mixed receptive/expressive language disorder, and 8% (n = 1) were diagnosed with attention deficit and hyperactivity disorder with expressive language disorder prior to enrollment. Seventy-seven percent (n = 10) of participants were non-Hispanic white (NHW), 15% (n = 2) were Hispanic (H), and 8% (n = 1) was biracial (B). Ages ranged from 3 years to 10 years with a mean of 6 years. Participants were categorized into two verbal ability groups, verbal and nonverbal, based on the Autism Diagnostic Observation Scale- 2^nd^ edition definitions for preverbal, phrase speech, and fluent speech [22]. Participants in the verbal group could speak at a four-year-old level in functional expressive language or higher, while the nonverbal group were either nonverbal (no words), preverbal (single words up to inconsistent use of simple phrases), or used phrase speech (regular production of non-echoed phrases made up of three independent units). Seventy percent (n = 9) were categorized in the verbal group, which included eight NHW and one B participants with ages ranging from 4 to 10 years. Of these, 78% (n = 7) were enrolled in speech language therapy, 78% (n = 7) were enrolled in occupational therapy, 56% (n = 5) were enrolled in physical therapy, 11% (n = 1) were receiving psychological therapy, 11% (n = 1) were receiving counseling, and 11% (n = 1) attended a developmental preschool. Twenty-two percent (n = 2) had received prior speech therapy, 22% (n = 2) had received prior occupational therapy, 11% (n = 1) had received prior physical therapy, 33% (n = 3) had received prior psychological therapy, 11% (n = 1) had received prior counseling, and 0% (n = 0) had priorly been enrolled in a developmental preschool. Thirty percent (n = 4) were categorized in the nonverbal group, comprised of two NHW and 2 H participants with ages ranging from 3 to 5 years. Of these, 75% (n = 3) were enrolled in speech language therapy, 50% (n = 2) were enrolled in occupational therapy, 25% (n = 1) were enrolled in physical therapy, and 25% (n = 1) attended a developmental preschool. None of the participants in the nonverbal group were receiving psychological therapy or counseling. The mean ASD severity per the Autism Diagnostic Scale, 2^nd^ edition (ADOS-2), comparison score in the nonverbal group was 7.66 (SD = 0.88, range) and the median was 8. The mean ASD severity ADOS-2 comparison score in the verbal group was 6.78 (SD = 1.01, range) with the median of 7. See Table 1 for summary statistics of demographic data and Fig 2 for participants’ prior therapy histories.

**Table 1:** Summary Statistics for Demographic Data.

	*n*	%
*Child race/ ethnicity*		
Non-Hispanic White	10	77%
Hispanic	2	15%
Biracial	1	8%
*Child Diagnosis*		
Autism	10	77%
Developmental delay with mixed expressive language disorder	2	15%
Attention deficit and hyperactivity disorder with expressive language disorder	1	8%
*Child biological sex*		
Male	12	92%
Female	1	8%
*Child age at referral (years)*		
Mean	5.85	
Standard deviation	2.48	
Range	3-10	
*Child verbality*		
Verbal	9	70%
Nonverbal	4	30%
*ADOS-2 comparison scores in Nonverbal Group*		
Mean	7.67	
Standard deviation	0.88	
Range	9−9	
Median	8	
*ADOS-2 comparison scores in Nonverbal Group*		
Mean	6.78	
Standard deviation	1.01	
Range	2-10	
Median	7	
*Current speech therapy enrollment*		
Verbal group	7	75%
Nonverbal group	3	78%
*Current occupational therapy enrollment*		
Verbal group	7	78%
Nonverbal group	2	50%
*Current physical therapy enrollment*		
Verbal group	5	56%
Nonverbal group	1	25%
*Current psychological therapy enrollment*		
Verbal group	1	11%
Nonverbal group	0	0%
*Current enrollment in counseling*		
Verbal group	1	11%
Nonverbal group	0	0%
*Current enrollment in developmental preschool*		
Verbal group	1	11%
Nonverbal group	1	25%
*Prior speech language therapy*		
Verbal group	2	22%
Nonverbal group	0	0%
*Prior occupational therapy*		
Verbal group	2	22%
Nonverbal group	0	0%
*Prior physical therapy*		
Verbal group	1	11%
Nonverbal group	0	0%
*Prior psychological therapy*		
Verbal group	3	33%
Nonverbal group	0	0%
*Prior enrollment in counseling*		
Verbal group	1	11%
Nonverbal group	0	0%
*Prior enrollment in developmental preschool*		
Verbal group	0	0%
Nonverbal group	0	0%

Summary of demographic and baseline clinical characteristics for study participants by group. Values are presented as number and percentage.

### Intervention

Demographic data; past, family, and social histories; and prior therapy evaluations were collected during the initial consultation for enrollment into the ASD program. Caregivers were given the option to enroll the child in the AAT study at the follow-up appointment after the diagnostic assessment was completed, or upon enrollment in the ASD program if the child had an ASD diagnosis. Three children who did not meet ASD diagnostic criteria were enrolled as they had been diagnosed with developmental delays, speech disorders, and behavioral concerns. Each AAT study lasted between eight to 12 weeks, and caregivers had the option to re-enroll the child for the next study to continue addressing behavioral concerns. Animal-assisted therapy sessions lasted 30 to 45 minutes, depending on the age and ability of the participant. Sessions started with five to 10 minutes of free play with the canine, followed by sensory integration therapy based on the child’s identified sensory profile. Participants then entered the clinic room for 20 to 30 minutes of a seated activity in which the therapist, a certified pediatric nurse practitioner (CPNP), taught coping and socialization strategies to participants and caregivers. Sessions concluded with five minutes of play with the canine. Play activities include fetch, soccer, hide and seek, and puzzles. The canine was trained to lay under the table in the clinic room while the therapist was working with the participants. He would provide grounding, light pressure, and deep pressure therapy if the participant became anxious during the sessions. The seated work was recorded using two cameras to ensure visibility of the interactions between the participant and therapist, canine, and caregiver; participant vocalizations and verbalizations; and participant facial expressions. Importantly, the play time was not recorded; therefore, the data only represents interactions and behavior occurring during the seated work in the clinic room.

The canine is a male black Labrador Retriever who was between one to two years old during this study. His handler is his owner and the CPNP who oversees the A4A program at the university and is the therapist during AAT sessions. He is certified as both a therapy and service dog and completed almost 200 therapy sessions during the study period. He learned 60 different commands to facilitate interaction with pediatric participants and could detect the onset of anxiety and emotional dysregulation. The canine provided deep pressure therapy and comfort measures when participants were anxious, assisted in grounding the participant during sessions, and provided an outlet for tactile stimulation by allowing participants to pet and brush his fur. In addition, the canine had learned 10 American sign language signs to promote interaction with nonverbal participants.

### Instruments

The *Observation for Human Animal Interaction for Research* (OHAIRE) Coding System from University of Arizona was used to note human bonding and prosocial behavior occurring during AAT sessions. Inter-rater (kappa = 0.81) and intra-rater (kappa = 0.87) reliability were obtained, and internal structure analysis demonstrated consistency with the OHAIRE tool and other social skill assessments [26]. Observable behavior categories captured by the OHAIRE Coding System include interactive behaviors (social communication and environmental interaction), emotional displays (facial and verbal), and interfering behaviors (aggression, overactivity, and isolation) [27]. Each member of the research team passed a certification exam to use the OHAIRE ethogram and interrater reliability was 0.773 and 0.775, comparable with moderate consistency between raters and close to strong consistency score of 0.817.

The second instrument used was the *3-AAT Observation* ethogram, adapted with permission from Johns Hopkins University, which was used to code both canine and human interaction during AAT sessions. The revised ethogram appraises the frequency of six domains of human interaction (affection, play, care, communication, comfort, withdrawal) and 24 subdomains of human behavior that can occur in an AAT session as seen in Table 2. The ethogram assesses the frequency, intensity, and duration of the interactions between the canine and child. Frequency is measured by a tally count and duration is assessed by rounding either up or down to the nearest 30 second mark. Each interaction has a code for intensity from 0 (not intense) to 3 (the most intense), and the mode of the interaction was entered to run the analysis.

**Table 2:** *3.AAT Observation* ethogram Conceptual Definitions.

Interaction Domains	Conceptual definition
Affection: Laughing	Child laughs while playing with dog
Affection: Petting during play	Child pets dog during play
Play: Fetch	Child plays fetch with dog
Play: Hide and Seek	Child plays hide and seek with dog
Play: Puzzles	Child plays puzzles with dog
Care: Brush	Child brushes dog’s coat
Care: Walking	Child walks down on leash
Communication: Name	Child says dog’s name
Communication: Command	Child says command
Communication: Verbal Affirmation	Child says verbal affirmation
Comfort: Head	Child touches top of dog’s head while seated
Comfort: Face	Child touches dog’s face
Comfort: Back	Child touches dog’s back
Comfort: Belly	Child touches dog’s belly
Comfort: Ears	Child touches dog’s ears
Comfort: Hugs	Child hugs dog
Comfort: Kiss	Child kisses dog
Comfort: Treat	Child gives dog a treat
Comfort: Floor	Child sits on floor beside dog
Comfort: Laying	Child lays on dog
Withdrawal: Moves away	Child moves away from dog
Withdrawal: Hides	Child hides from dog
Withdrawal: Crying	Child cries when dog is present
Withdrawal: No!	Child says “No!” to interacting with dog

This table outlines the conceptual definitions for each domain and subdomain included in the *3-AAT* (Animal-Assisted Therapy) *Observation* ethogram used to code human and animal interaction occurring during animal-assisted therapy sessions. Definitions are provided to clarify the behavioral and interactional constructs assessed during observation, supporting consistency in coding and interpretation.

The *Strengths and Difficulties Questionnaire* (SDQ) is a 25-item tool developed to assess five domains: *Emotional Problems*, *Conduct Problems*, *Hyperactivity*, *Peer Problems*, and *Prosocial*. The SDQ is the most used instrument in measuring therapeutic effect of interventions within the emotional state in children and adolescents ages 2–18 years, demonstrating reliable internal consistency and validity [28]. The SDQ has two versions, one to be administered at the initiation of therapy and then a follow-up version that includes two additional questions examining the impact of the therapeutic intervention. Scores from four behavioral domains range from zero (“normal”) to 10 (“very high concerns”), while *Prosocial* domain scores are reversed so that 10 indicates “normal” behavior and zero signifies “very high concerns”. The SDQ also assesses the total, impact, internalizing, and externalizing scores to provide a complete overview of the child’s behavior during the past four weeks. Total scores are the sum of the behavior domains except the *Prosocial* domains and range from zero (normal) to 40 (very high). *Impact* scores range from zero (normal) to 10 (very high) and reflect overall distress and impairment interfering with daily social interactions. *Internalizing* scores reflect the *Emotional Problems* and *Peer Problems* domains, while *Externalizing* scores are the sum of the *Conduct Problems* and *Hyperactivity* domains. Both the *Internalizing* and *Externalizing* domain scores range from zero (normal) to 20 (very high). The initial SDQ was administered at the beginning of the study and the follow-up assessment was used subsequently every four weeks until the study concluded.

Lastly, the *Positive and Negative Affect Schedule* (PANAS) was administered weekly to measure therapeutic effects occurring in everyday life outside of the AAT session. The PANAS is a 20-item questionnaire utilizing a 5-point Likert scale to assess positive and negative affect occurring the week prior to administration. Each domain has 10 items with scores ranging from 10 to 50, with lower scores reflecting lower levels of positive or negative affect and higher scores signifying higher positive or negative affect [29]. Total scores on each scale are obtained by adding the scores for each item. Analysis of the PANAS demonstrates very good internal consistency reliability, with alphas ranging from 0.86 to 0.90 for *Positive* affect and from 0.84 to 0.87 for *Negative* affect [30,31]. The PANAS questionnaire is used for children over the age of 6 years, therefore, six participants were excluded from the weekly analysis.

## Results

### RQ1. Human-Animal interaction

A total of 2,071 interactions were analyzed with the *3-AAT Observation* ethogram to measure affection, play, communication, comfort, withdrawal, and care behaviors, and 210 interactions were analyzed using the OHAIRE assessment to measure social communication, interaction, overactivity, and interfering behaviors. Both assessments noted the same behaviors, strengthening the veracity of the analysis. However, as previously stated, the seated portion was the only part of the session able to be recorded, limiting some of noted interaction between participants and the canine.

The OHAIRE *Social Communication* category showed a significant difference between the groups (*t*(270)=−3.29, *p* = .002, *d* = −0.50). The verbal group (M = 3.96, SD = 2.14) demonstrated significantly more frequencies in social communication behaviors than the nonverbal group (M = 2.92, SD = 2.25) at α = 0.1 level. Specifically, the social communications with the therapist were reported more in the verbal group (M = 3.75, SD = 2.15) than the nonverbal group (M = 2.03, SD = 1.92), which indicated the statistically significant differences between two groups (*t*(101.70)=−5.92, *p* < .001, *d* = −0.82). Conversely, the nonverbal group demonstrated more social communication with the caregiver (M = 1.86, SD = 2.19) than the verbal group (M = 1.00, SD = 1.35), which was statically significant (*t*(70.69)=2.90, *p* = .005, *d* = 0.55).

Analysis of the OHAIRE *Environmental Interaction* domain showed the verbal group (M = 3.56, SD = 2.38) interacted more with the environment, which includes the canine, more than the nonverbal group (M = 2.39, SD = 2.24). This difference is statistically significant (*t*(270)=−3.39, *p* < .001, *d* = −0.50). The interactions with the canine were reported more in the verbal group (M = 2.46, SD = 2.30) than the nonverbal group (M = 1.37, SD = 1.66), which was statistically significant (*t*(126.54)=−4.08, *p* < .001, *d* = −0.50). However, *t*he average OHAIRE scores of interactions with objects in the room, such as other toys, were not statistically significantly different between the verbal and nonverbal groups.

**Fig 3:**
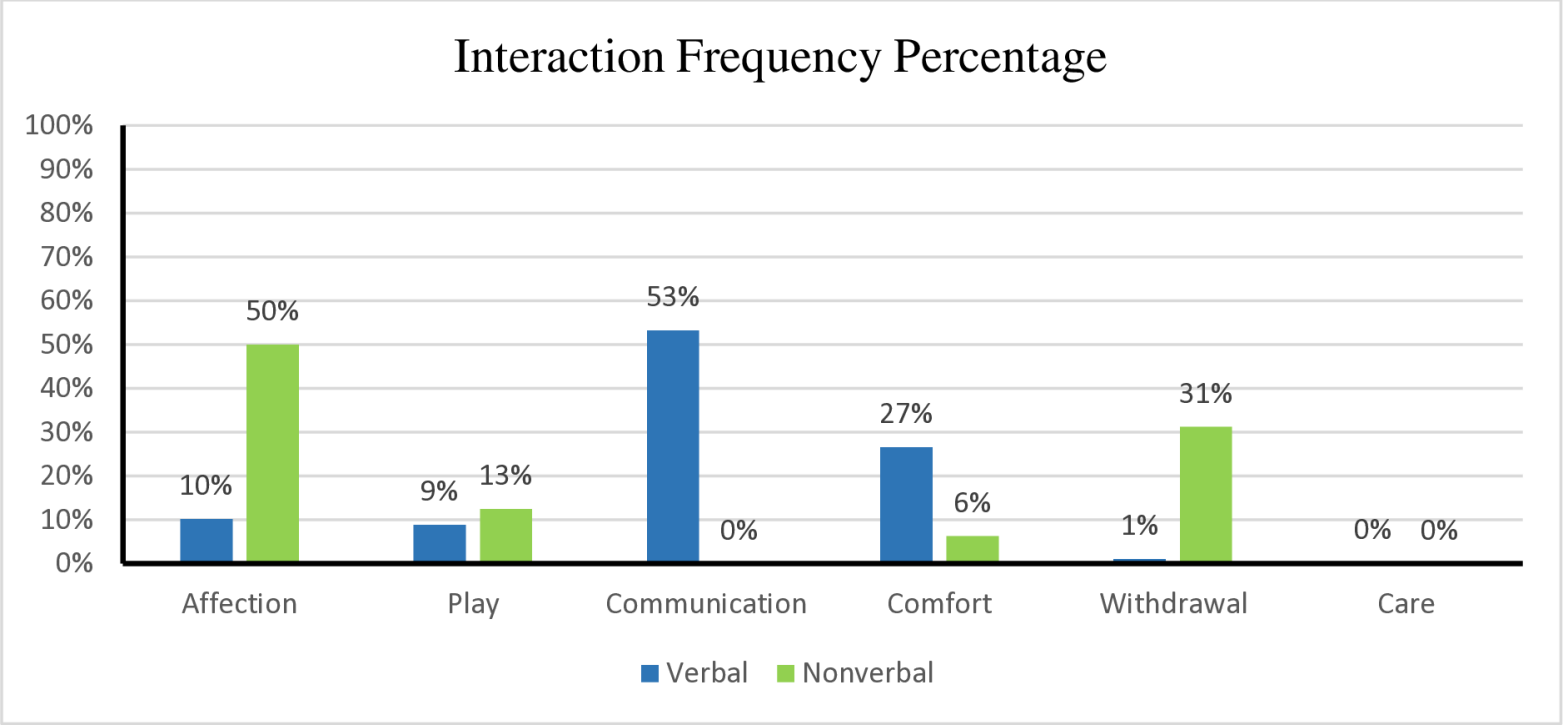
Percentage of interaction of *3-AAT* domains among groups. (A) This figure illustrates the percentage of interaction between the domains of the *3-AAT* (Animal-Assisted Therapy) *Observation* ethogram across different participant groups. The data highlights how each group engages with the canine, with percentages indicating the interaction level per domain. Differences among groups reflect potential variation in domain relevance or influence. The verbal group had more minutes of interaction (909.5 minutes) compared to the nonverbal group (31 minutes), as depicted in Fig 4, which was also statistically significant (t(2)=2.01, p = 0.0014, df = 46). Interestingly, the mode for intensity for both groups was 2, indicating all participants interacted with the same interest, concentration, and strength while interacting with the canine, even though the nonverbal group interacted with the canine less than the verbal group. See Fig 4 for the percentage of interaction duration between the two groups.

Similar results were noted in the *3-AAT* ethogram analysis. A total of 2,007 interactions were coded in the verbal group, compared to 64 coded interactions in the nonverbal group. The verbal group had significantly more interactions with the canine than the nonverbal group at α = 0.1 level, and this difference was also statistically significant (*t*(89.47)=5.03, *p* < .001 with large effect size, *d* = 0.65). See Fig 2 for the frequency of canine interactions among groups. Notably, 50% of the interaction between the canine and the nonverbal group corresponded to the category of *Affection* when the child petted the canine while playing, compared to verbal group (10%), whose most used category of interaction was verbal *Communication* (53%). While 31% of the interaction among the nonverbal group corresponded to the *Withdrawal* category, analysis showed most of the withdrawal interaction occurred with one participant only who said “*No!*” frequently, even when he was not interacting with the canine, negatively skewing the results. See Fig 3 for interaction percentages among the *3-AAT Observation* ethogram domains.

**Fig 4:**
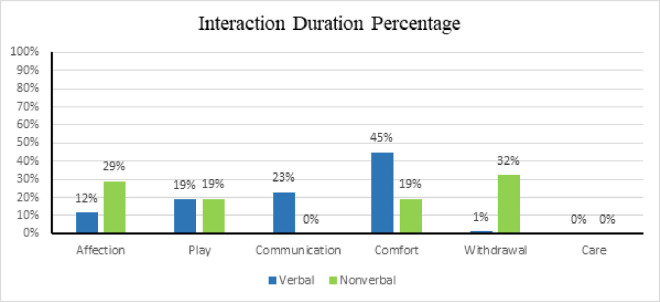
Percentage of interaction duration of *3-AAT* domains among groups. (A) This figure displays the percentage distribution of interaction duration across the domains of the *3-AAT* (Animal-Assisted Therapy) *Observation* ethogram for different participant groups. Each bar with associated percentages represents the percentage of time spent engaging with the canine, allowing comparison of interaction patterns across groups.

**Fig 5:**
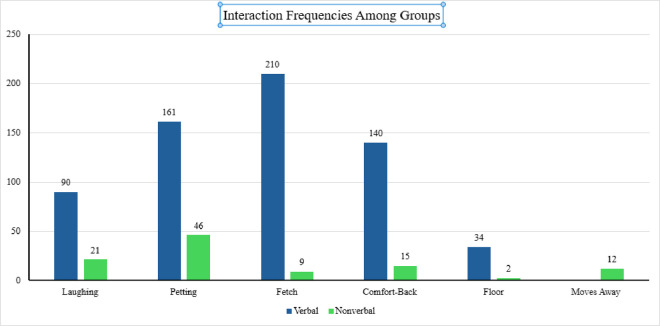
Interaction frequencies of *3-AAT* subdomains among group. (A) This figure illustrates the frequency of interactions within the subdomains of the *3-AAT* (Animal-Assisted Therapy) *Observation Form* ethogram among participant groups. Each bar with associated data points represents the total number of interactions per subdomain, allowing comparison of interaction patterns between groups.

*Hide and Seek, Puzzle, Walking, Name, Command, Verbal affirmation, Comfort-head, Comfort-face, Comfort-back, Comfort-belly, Comfort-ears, Hugs, Kiss,* and *Laying* interactions were not observed in nonverbal group. *Brush, Hides,* and *Crying* interactions were not observed in either group, and *Treat* (*N* = 1) and *Comfort-floor* (*N* = 2) interactions did not occur frequently enough in the nonverbal group to be able to calculate a difference between the groups. Thus, the frequencies of six interactions were analyzed (*Laughing, Petting, Fetch, Comfort-Back, Floor,* and *Moves away*) among the groups. A significant difference was noted between groups with the frequency of the *Fetch* interaction (*t*(46.95)=3.512, *p* < .001, *d* = 0.58), with the verbal group (M = 4.12, S.D. = 4.73) engaging in *Fetch* more than the nonverbal group (M = 1.50, S.D. = 0.84). However, *Laughing, Petting*, *Comfort-Back, Floor,* and *Moves away* interactions did not show significant differences in frequencies between the groups, as seen in Table 3. Fig 5 represents the total number of interactions between participants and the canine.

**Table 3:** Independent *t*-test Results for Interaction Frequency of *3-AAT* Subdomains Among Groups.

Interaction	N	M	S.D.	t-stat.	df	p-value	*d*
**Laughing**				−.14	42	.89	−.06
Verbal	90	2.50	2.16				
Nonverbal	21	2.63	2.62				
**Petting**				−.827	6.31	.44	−.62
Verbal	161	3.66	3.70				
Nonverbal	46	6.57	9.20				
**Fetch**				3.512	46.95	<.001	.58
Verbal	210	4.12	4.73				
Nonverbal	9	1.50	0.84				
**Comfort-Back**				−.503	2.04	.66	−.65
Verbal	140	2.98	2.82				
Nonverbal	15	5.00	6.93				
**Floor**				.57	18	.57	.43
Verbal	34	1.89	2.14				
Nonverbal	2	1.00	0.00				
**Moves Away**				−.097	17	.92	−.05
Verbal	20	1.67	0.99				
Nonverbal	12	1.71	1.11				

Results of independent *t*-tests comparing the frequency of interaction across *3-AAT* (Animal-Assisted Therapy) *Observation Form* ethogram subdomains between groups. Values are presented as mean ± standard deviation (SD) for each group, along with *t*-statistics, degrees of freedom (df), and *p*-values indicating statistical significance.

### RQ2. Prosocial behavior and emotional regulation within session

**Fig 6:**
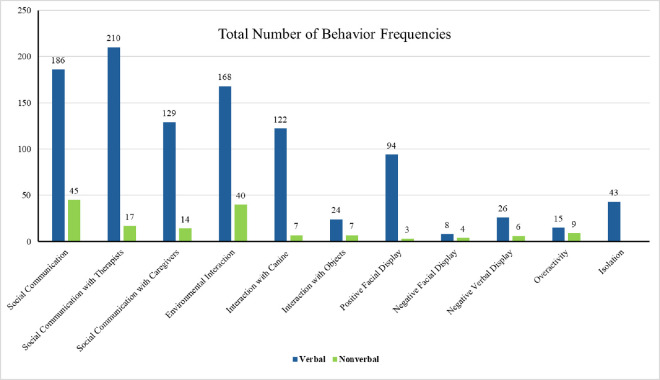
Behavior frequencies of OHAIRE scores among groups. (A) This figure depicts the frequency of observed behaviors as measured by the *Organization for Human-Animal Interaction Research and Education* (OHAIRE) ethogram across different participant groups. Each bar with associated data points represents the total frequency of specific behaviors, allowing comparison of behavioral patterns among groups.

The last two categories of the OHAIRE assessment (*Emotional Displays*, and *Interfering Behaviors*) were evaluated to answer RQ2. The *Emotional Displays* category contains two subsets (*Facial Display* and *Verbal Display*) with each having a positive and negative display, while the *Interfering Behaviors* category has three subsets (*Aggression*, *Overactivity*, and *Isolation*). However, the categories of positive verbal display and aggression were excluded from the data analysis because of zero sample size. There was a significant difference in *Overactivity* between the two groups (*t*(66.81)=3.07, *p* = .003, *d* = 0.64). The verbal group (M = 0.21, S.D. = 0.74) showed less overactive behaviors than the nonverbal group (M = 0.80, S.D. = 1.42). Similarly, the verbal group (M = 0.91, S.D. = 1.59) showed less *Isolation* behaviors than the nonverbal group (M = 3.59, S.D. = 2.42), which was also statistically significant and had a large effect size (*t*(72.40)=8.06, *p* < .001, *d* = 1.49). However, the four subcategories of *Emotional Display* behaviors did not show any significant difference between verbal and nonverbal groups, as depicted in Table 4 and Fig 6.

**Table 4:** Independent t-test Results for OHAIRE Scores Among Groups.

Behavior	N	M	S.D.	t-stat.	df	p-value	*d*
**Social Communication**				−3.29	270	0.002	−0.50
Verbal	186	3.96	2.14				
Nonverbal	45	2.92	2.25				
**Social Communication with Therapists**				−5.92	101.70	<.001	−0.82
Verbal	210	3.75	2.15				
Nonverbal	17	2.03	1.92				
**Social Communication with Caregivers**				2.90	70.69	0.005	0.55
Verbal	129	1.00	1.35				
Nonverbal	14	1.86	2.19				
**Environmental Interaction**				−3.39	270	<.001	−0.50
Verbal	168	3.56	2.38				
Nonverbal	40	2.39	2.24				
**Interaction with Canine**				−4.08	126.54	<.001	−0.50
Verbal	122	2.46	2.30				
Nonverbal	7	1.37	1.66				
**Interaction with Objects**				−1.09	104.31	.28	−0.15
Verbal	24	1.53	2.35				
Nonverbal	7	1.19	2.05				
**Positive Facial Display**				0.28	81.12	0.78	0.05
Verbal	94	0.84	1.22				
Nonverbal	3	0.90	1.48				
**Negative Facial Display**				0.85	77.06	0.2	0.15
Verbal	8	0.04	0.19				
Nonverbal	4	0.07	0.25				
**Negative Verbal Display**				1.54	59.42	1.28	0.40
Verbal	26	0.02	0.15				
Nonverbal	6	0.17	0.72				
**Overactivity**				3.07	66.81	0.003	0.64
Verbal	15	0.21	0.74				
Nonverbal	9	0.80	1.42				
**Isolation**				8.06	72.40	<.001	1.49
Verbal	43	0.91	1.59				
Nonverbal	7	3.59	2.42				

This table presents the results of independent *t*-tests comparing the *Organization for Human-Animal Interaction Research and Education* (OHAIRE) ethogram scores among participant groups. Values are reported as mean ± standard deviation (SD) for each group, along with the *t*-statistic, degrees of freedom (df), and corresponding *p*-values to indicate statistical significance.

### RQ3. Prosocial behavior and emotional regulation apart from sessions

The monthly SDQ and weekly PANAS scores reflected the effect of AAT on behavior during daily life activities apart from AAT sessions. Unfortunately, caregivers of the four children in the nonverbal group and two in the verbal group did not complete the SDQ assessment consistently, rendering their results invalid. Likewise, the PANAS assessment is normed for children ages 6 years and older; therefore, it was not used to assess the weekly behavior of the four children in the nonverbal group. Therefore, the following aggregate data reflects the mean scores of the SDQ and PANAS for the verbal group only.

**Fig 7:**
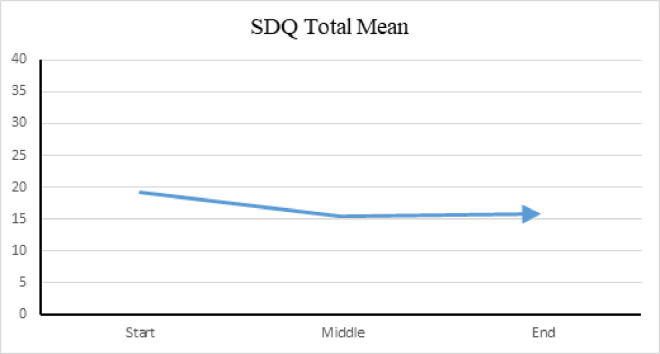
SDQ total mean among verbal group. (A) This figure shows the mean total score on the *Strengths and Difficulties Questionnaire* (SDQ) for participants in the verbal group. The total score reflects the overall level of emotional and behavioral difficulties experienced by participants in the week prior to AAT sessions at the beginning, middle, and conclusion of the study.

SDQ *Total* mean scores (range zero to 40) reflect overall prosocial behavior and emotional regulation demonstrated by participants for the preceding month. Lower scores indicate improvement in behavior. The SDQ mean scores show improvement in all areas by the midpoint of the study, with stabilization of behaviors noted in the last half of the study. See Fig 7 for SDQ *Total* scores.

**Fig 8:**
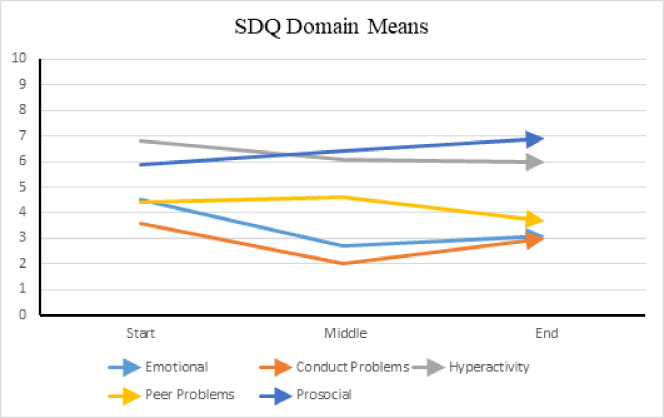
SDQ domain means among verbal group. (A) This figure presents the mean scores for each domain of the *Strengths and Difficulties Questionnaire* (SDQ) within the verbal group. The domains assessed include Emotional Symptoms, Conduct Problems, Hyperactivity/Inattention, Peer Relationship Problems, and Prosocial Behavior. The mean scores represent the overall level of emotional and behavioral difficulties experienced by participants during the week prior to AAT sessions at the beginning, middle, and end of the study. For all domains except Prosocial Behavior, lower scores indicate improvement. In contrast, higher scores in the Prosocial domain reflect positive behavioral change.

Similarly, SDQ domain scores (range zero to 10) exhibited improvement by the midpoint of the study. See Fig 8 for SDQ domain scores. Since the *Prosocial* domain scores are inverse, an increase in scores reflects overall improvement in behavior while the other four domains should note a decrease in scores. Also, the *Conduct Problems* and *Emotional Problems* domains showed a slight increase in behaviors after the midpoint of the study.

**Fig 9:**
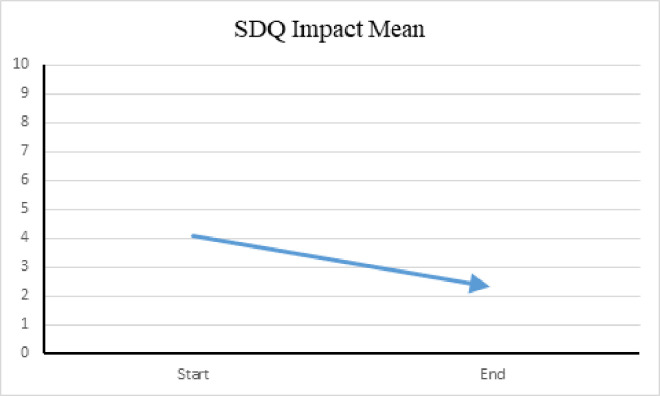
SDQ impact mean among verbal group. (A) This figure illustrates the mean Impact score from the *Strengths and Difficulties Questionnaire* (SDQ) for participants in the verbal group. The Impact score reflects the perceived distress and social impairment associated with emotional and behavioral difficulties at the beginning and conclusion of the study. The downward arrow indicates improvement.

SDQ *Impact* scores range from zero to 2 (not at all [0], only a little [0], a medium amount [1], a great deal [2]) to a total impact score of 10 and reflect the overall distress and impairment reported by caregivers for the preceding month. Distress and impairment are measured by whether they upset or distress the child, interfere with home life, friendships, classroom learning, or leisure activities. The lower *Impact* scores indicate the child experienced less distress or impairment during the preceding month, which is noted in Fig 9.

**Fig 10:**
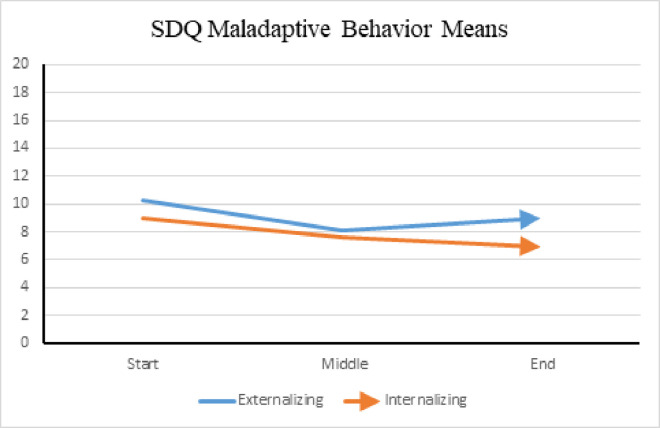
SDQ maladaptive behavior means among verbal group. (A) This figure displays the mean scores for maladaptive behaviors, derived from relevant subscales of the *Strengths and Difficulties Questionnaire* (SDQ), among participants in the verbal group. Higher scores indicate greater levels of maladaptive behavior. Improvement in both the Externalizing and Internalizing domains was noted; however, more improvement was noted with Internalizing behaviors. Comparably, the means of the PANAS weekly score, as portrayed in Fig 11, reflect improvement in behavior and emotional regulation in the verbal group, with an increase in positive affect and a decrease in negative affect.

Maladaptive behavior is depicted in the *Externalizing* and *Internalizing* domains. Scores range from zero to 20, with lower scores indicating less occurrence. *Externalizing* scores are comprised of the *Conduct Problems* and *Hyperactivity* domains, while *Internalizing* scores are the sum of the *Emotional Problems* and *Peer Problems* domains. Fig 10 shows improvement in the *Internalizing* domain and a slight uptick in the *Externalizing* domain, most likely influenced by the *Conduct Problems* domain score.

**Fig 11:**
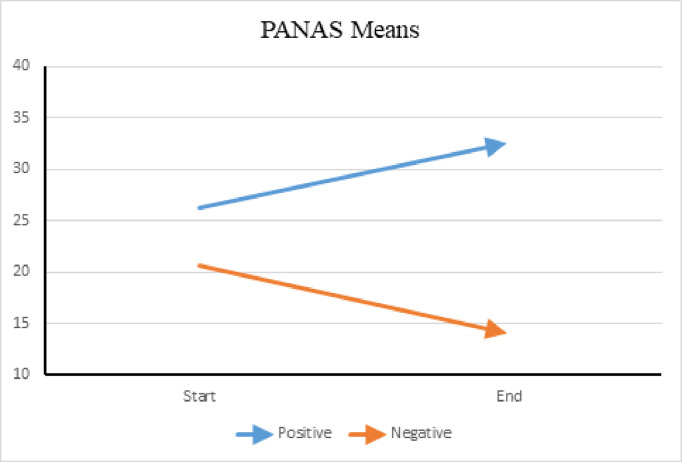
PANAS Means Among Verbal Group. (A) This Figure presents the average scores for Positive Affect and Negative Affect, as measured by the *Positive and Negative Affect Schedule* (PANAS), within the verbal group. These scores represent participants’ self-reported emotional states. Higher Positive Affect scores indicate an increase in positive emotions and behaviors, while lower Negative Affect scores suggest a reduction in negative emotions and behaviors. The data show improvements in both affect domains.

## Discussion

Results of this pilot study indicate that mental health practitioners can use trained canines to promote prosocial behavior and emotional regulation and enhance therapeutic outcomes for children with ASD regardless of verbality. Both human-animal interaction ethograms demonstrated increased prosocial behavior and emotional regulation within the AAT sessions, while both the SDQ and PANAS mirrored this effect apart from the AAT sessions for the verbal group. Furthermore, the aggregate analysis showed a cumulative progression of desired behavior, supporting evidence for the long-term beneficial effects of AAT.

Social communication was apparent during the recorded sessions, with children in the verbal group communicating mostly with the therapists and children in the nonverbal group directing nonverbal communication and phrase speech toward their caregiver. Both groups interacted with the canine with the same intensity; however, children in the verbal group interacted with the canine more than the nonverbal group. This was mostly seen in verbal interaction noted on the OHAIRE ethogram. Yet, the *3-AAT Observation* ethogram showed that children in the nonverbal group demonstrated more *Affection* behaviors that did not require verbalization with the canine, and both groups displayed *Play* and *Comfort* behaviors with the canine. Likewise, there were no differences in negative verbal displays and facial expressions between groups, indicating both groups enjoyed interacting with the canine.

The OHAIRE ethogram noted increased frequency in *Overactivity* and *Isolation* behaviors among the nonverbal group; however, there was not a difference in *Interfering* behaviors between the groups. These results concur with prior research noting the connection between verbality and emotional regulation [1,4]. Current recommendations for children with externalizing behaviors include addressing language deficits [4], and AAT may be an avenue to increase verbality among nonverbal children with ASD.

The SDQ scores reflected an improvement in prosocial behavior and emotional regulation apart from AAT sessions in the verbal group with a noted plateau after the midpoint of the study. The *Emotional Problems* and *Conduct Problems* domains were identified as the most prevalent behavioral concerns at the of the study. These scores affected the *Externalizing* behaviors score, which demonstrated the same uptick at the midpoint of the study. However, the SDQ *Impact* scores decreased throughout the study, implying participants experienced less distress and impairment at home, school, with friendships, and in leisure activities. Moreover, the weekly PANAS scores reflected more positive behaviors and less negative behaviors at the end of the study, agreeing with the monthly SDQ assessments.

One focus of future research in AAT is to determine the influence of the canine on the therapeutic outcomes in participants [32–34]. Also, possible placebo effects should be analyzed. In our study, RQs 1 and 2 possible placebo effects are minimal since these questions measure the child’s interaction and bonding with the canine. Further investigation of possible placebo effects for RQ3 is warranted, as the child may experience improvements in behavior due to the positive relationship with the therapist and not because the canine was present.

As previously stated, the nonverbal group was comprised of younger participants (ages 3to 5 years) while the verbal group contained a broader range of participants (ages 4 to 10). A larger sample size with older nonverbal children is recommended to see if nonverbal children of all ages have more interactions in the *Affection* domain than those who are verbal. Both groups were similar in that they had participants who were receiving speech language and occupational therapies at the time of the study and each contained one participant enrolled in a developmental preschool. None of the participants in the nonverbal group had received prior psychological therapy or counseling due to their young age; however, they were enrolled in allied health therapies, giving them some experience in a therapeutic setting. Therefore, we consider the data for RQs 1 and 2 to accurately reflect behavior occurring within a typical therapy session. Likewise, one participant in the verbal group saw a school counselor during the school year, one other participant was seeing a psychologist at the time of this study, and one participant had seen a psychologist for therapy prior to enrolling in the study. Most of the participants in the verbal group had not seen a therapist to address behavioral concerns, equating their therapeutic experiences to those in the nonverbal group. Lastly, the mean ADOS-2 comparison score for the nonverbal group (7.66) and the verbal group (6.78) were similar, meaning participants from both groups displayed moderate to severe symptoms of ASD. However, due to the smaller sample size, we could not run an analysis to confirm there was not a statistical difference in ASD symptomology between the groups.

Future studies should enroll a large enough sample to conduct more advanced statistical models, such as using a structural equation modeling analysis to investigate significant differences in the interaction between therapy animal and autistic children, prosocial behavior and emotional regulation regarding child’s verbal ability, race, and/or cultural backgrounds. Likewise, while our study expanded the outcomes to include effects experienced in-between AAT sessions, future studies should examine the lasting effect of AAT months after the sessions have concluded to compare results of AAT with the long-term outcomes experienced by participants receiving traditional therapy modalities. Furthermore, the possible influence of canine interaction on participants’ oxytocin levels should be assessed to see if the oxytocin levels increase during therapy and remain elevated after AAT has ended [35,36].

Other limitations of this study include omission of the PANAS and SDQ scores in the nonverbal group did not allow for comparison of the behavior apart from AAT sessions. The use of one canine is another study limitation. Also, not all AAT sessions were recorded due to technical problems with the overhead camera and handheld camera. Interactions occurring outside of the clinic room were not recorded and could not be coded or evaluated; therefore, interactions with the canine such as *Hide and Seek* and *Walking* were not included in the analysis.

## Conclusion and recommendations

Results from this study indicate mental health practitioners can successfully incorporate canines into the plan of care for children with ASD to achieve therapeutic outcomes. Furthermore, AAT may be an option for families who cannot afford a service canine of their own. Future research must investigate the impact of canines in AAT sessions, determining if canines only aid the therapy process or can be trained to perform activities to enhance health outcomes that cannot be attained without them. One hypothesis explaining the mechanism of action for AAT sessions indicates that the human-animal bond is a vital part of the therapeutic process and desired outcomes would not be attained without the inclusion of the animal in therapy sessions [32,33]. This premise is supported by research demonstrating the use of live canines over plush toys or other objects [15]. The results also support further investigation into AAT activities to promote communication among autistic children with limited verbality, reduce emotionality, and increase socialization. Mental health practitioners can use canines as “transitional objects” in AAT sessions, allowing children with ASD to practice social skills in AAT sessions and later apply learned behavior in everyday settings [37]. Thus, activities to achieve desired outcomes must be investigated to standardize therapy sessions so all mental health practitioners obtain expected results. Furthermore, human-animal interaction must be investigated among ethnic groups, ages, genders, intellectual abilities, social motivation, developmental level, and verbal abilities to determine best practice for utilizing canines to assist the pediatric ASD population. Also, the ethical use of canines is an important subject for future studies and should investigate the number and duration of daily sessions, rest requirements between sessions, and the best canine temperament for this type of work. Lastly, the effect of canine interaction on human cortisol and oxytocin levels could explain the documented success of AAT [35,38], and merits further exploration. The canine was trained to provide comfort measures and deep pressure upon detecting the escalation of stimming behavior or anxiety in the participants as these techniques naturally increase human oxytocin levels. These interventions could have resulted in improved socialization and emotional regulation, hypothesizing that AAT could be used to elevate endogenous serum oxytocin levels in children with ASD [38,36]. Nonetheless, current literature supports the incorporation of canines in mental health therapy sessions for pediatric patients with ASD to achieve therapeutic outcomes.

## Supporting information

S1 AAT Verbality IRB Protocol 1. (PDF)

S2 AAT Verbality IRB Protocol 2. (PDF)

S3 AAT Verbality TREND Checklist. (PDF)
