# Supplementary material for: The effect of Animal-assisted therapy on prosocial behavior and emotional regulation in autistic children with varying verbal abilities: A pilot study
Source: PLoS One. 2025 Jul 1;20(7):e0326085. doi: 10.1371/journal.pone.0326085 (PMC12212493; doi:10.1371/journal.pone.0326085)
Supplement: S1 — (PDF) [file pone.0326085.s001.pdf]

University of Arkansas System

Document Overview
Description: Animal-Assisted Therapy in Autism Care Management
Explanation:
Organization Doc Num:

Protocol Summary
Protocol Number: 2201382953
Sequence Number: 6
Status: Active - Open to Enrollment
Expiration Date: 03/31/2024
Last Approval Date: 04/01/2023
Investigator: Michele R Kilmer

Protocol Details
Type: Expedited
Summary/Keywords:
Application Date: 02/07/2023
Reference ID1:
Reference ID2:
FDA Application No:
Title: The Effect of Animal-Assisted Therapy on Prosocial Behavior in Children with Developmental Delay or Autism Spectrum Disorder

Areas of Research

Code	Description
000001	All Research Areas

Organizations

Type	Organization	Address
Performing Organization	University of Arkansas	University of Arkansas 1125 West Maple Street 316 ADMN Bldg, Fayetteville, AR 72701 USA

Funding Source

Type	Number/Code	Source	Title
Internally Funded/ Unfunded Research	unfunded	N/A	

Subjects

Subject	Count
Children	20
Adults	40

Investigators

Person Name: Michele R Kilmer
Role: Principal Investigator
Units: CC012723 UAF | NURS | Department of Nursing
Affiliation: Supervisor
Office Phone: 479-575-5466
Mobile:
Email: michelek@uark.edu

Person Name: Lauren B Quetsch
Role: Co-Investigator
Units: CC012702 UAF | PSYC | Psychology
Affiliation: Supervisor
Office Phone: 479-575-5817
Mobile:
Email: quetsch@uark.edu

Study Personnel

Person Name	Role	Affiliation	Email
Emily Ann Meade	Study Personnel	Student Investigator	eameade@uark.edu
Brooke A. Weaver	Study Personnel	Student Investigator	baw042@uark.edu
Emma B. Mitchell	Study Personnel	Student Investigator	ebmitche@uark.edu
Madelyn N. Talbert	Study Personnel	Student Investigator	mntalber@uark.edu
Rebecca S Bradley	Study Personnel	Student Investigator	rsb009@uark.edu
Harlee Onovbiona	Study Personnel	Student Investigator	honovbio@uark.edu
Emily-Anne Del Rosario	Study Personnel	Student Investigator	esdelros@uark.edu
Emily Shah	Study Personnel	Student Investigator	eshah@uark.edu
Emily Gail Grant	Study Personnel	Student Investigator	eggrant@uark.edu
Jewel Marie Hernandez	Study Personnel	Student Investigator	jmh107@uark.edu
Sarah Margaret Huetter	Study Personnel	Student Investigator	smhuette@uark.edu

Questionnaire

**Questionnaire Name:**	Human Subjects Protocol Interview		
**Description:**	Human Subjects Protocol Interview		
**Module:**	IRB	**Sub Module:**	
**Protocol Number:**	2201382953	**Sequence Number:**	6
**Principal Investigator:**	Michele R Kilmer		
**Title:**	The Effect of Animal-Assisted Therapy on Prosocial Behavior in Children with Developmental Delay or Autism Spectrum Disorder		

- **What is the purpose of this research? Please explain both why you are doing the research (class assignment, thesis, etc.) AND/OR state your hypothesis. See attachment is not a sufficient response.**
  The proposed project will investigate the effects of utilizing animal-assisted therapy (AAT) in the therapy plan for youth with autism spectrum disorder (ASD) or developmental delay (DD). The purpose of this study is to explore identified gaps in knowledge pertaining to AAT in pediatric ASD and DD care management. Specifically, we seek to document canine care and behavior during training and AAT sessions, assess human and canine interaction during AAT sessions, and analyze long-term effects of AAT on social-emotional and adaptive functioning in the pediatric participants once the sessions have ended. This study will address the following research questions: 1. What measures should be taken to ensure protection of the canine before, during, and after AAT sessions as well as during training? 2. What human-animal interaction behaviors are present during AAT sessions with pediatric participants ages 12 months to 18 years? 3. What effect does AAT have on pediatric development and social-emotional and adaptive functioning? 4. Are effects in development and social-emotional and adaptive functioning produced by AAT sustainable after completion of the 12-week AAT program?

- **Are you collecting data about living individuals?**
  Yes

  - **Are you collecting data through intervention or interaction with these individuals?**
    Yes

    - **Beyond the basic Participant Types (children, UofA Students, adults, etc.) named elsewhere in this application, do you have a target population (particular group of people) you want to recruit? Some examples might be students in a particular class, members of a particular group or network, people in a specific age range (whether adult or minor), children in a particular school or class, etc.**
      Yes

      - **Describe your target population.**
        Dr. Kilmer collaborates with the Community Clinic of NWA who refer children, ages 18 months to 18 years, at risk for developmental delay or autism spectrum disorders to her clinic, Access for Autism (A4A), for further developmental evaluation and care management. Children receiving care management by the A4A program who have developmental or behavioral concerns can volunteer to enter this study.

    - **How are you recruiting participants? Are you standing in a public place asking people to take a survey, sending out introductory emails, posting an ad or blurb on a website or social media, posting a flyer in a public location, etc.? **Please note that all recruitment materials will need to be uploaded in the Notes and Attachments section.**

Dr. Kilmer will discuss the study with parents/guardians whose children have been referred to the A4A clinic for further developmental evaluation. Participation in the study is voluntary and does not affect other services provided by the A4A clinic.

- **Provide a brief description of the procedures involving the participants.**

  All interactions between the canine and participants will occur either at the A4A clinic, in the EPLEY Center for Healthcare Professionals, or at the Psychology lab on campus. The first interaction between the canine and the participant will focus on introducing the two and facilitating a bond through play, such as petting or brushing the canine, or throwing a ball. The Purdue University Veterinary College of Medicine O'HAIRE Coding System will be used to observe and code human behavior during this session. Drs. Kilmer and Quetsch use a variety of behavioral and developmental evaluations to identify concerns and provide strategies for home therapy to improve delays: 1. Participants ages 8 years and older who are cognitively capable will be asked to self-report their emotional state before and after each AAT session using an 11-point Likert scale chart in the shape of a thermometer, with ranges from "no distress/totally relaxed" to "highest anxiety/stress that you've ever felt." 2. Strengths and Difficulties Questionnaire 3. Positive and Negative Affect Schedule 4. Ages and Stages Questionnaire- 3rd edition 5. Ages and Stages Questionnaire: Social and Emotional - 2nd edition 6. Social-Emotional Assessment Measure 7. Vineland- 3rd edition 8. Social Responsiveness Scale- 2nd edition 9. PEDS: DM-AL 10. Standford-Binet Intelligence Quota Test Caregiver satisfaction and perspectives of AAT integration in therapy sessions will be assessed via surveys. Demographic data including participant age, gender, race/ethnicity, referral source, prior medical diagnoses, age of ASD or DD diagnosis, medications, and prior allied health or psychological therapies will be collected on all participants. Dr. Kilmer will incorporate AAT into the therapy sessions at the A4A clinic that are guided by the Ages and Stages developmental and social/ emotional assessments to target gross and fine motor skill, language, social, and emotional development. Parents will be given handouts that are designed to address deficits identified by the ASQ-3, ASQ:SE-2, and SEAM assessments. The canine will be present to interact with children as they are learning their skill for that week. For example, the canine will interact with the participant while the participant is learning a new skill, such as improvement in handwriting, and the participant will be able to interact and play with the canine if the participant focuses on completing the therapy assignment. Likewise, participants will earn points if they perform their therapy strategies at home and can use those points to interact and play with the canine at their next therapy session. Plush dog toys who look like the canine will be given to pediatric participants as incentive to perform their home therapies. The following data will be collected on the canine: 1. The Canine Behavioral Assessment and Research Questionnaire (C-BARQ), a validated measure to quantify animal behavior during training developed by the University of Pennsylvania in 2003. The C-BARQ database will be used to compare the canine's training progress with other Black Labrador Retriever dogs and can also alert to the onset of behavioral problems so Dr. Kilmer can target these concerns early in the training process. 2. The canine behavior ethogram which assesses 26 canine behaviors during human-animal interactions. The ethogram is divided into three categories: affiliative indicators, moderate stress indicators, and high-stress indicators. The research team will track the number of AAT sessions in which the canine participates each day to assess for variations in temperament and performance. 3. The exact minutes in which canine behaviors are present will be recorded in each AAT session, noting patterns that indicate fatigue or distress. Human-animal interaction will be assessed by: 1. The Purdue University Veterinary College of Medicine O'HAIRE Coding System will be used to observe and code human behavior during AAT sessions. Observable behaviors categories captured by the OHAIRE Coding System include Interactive Behaviors (Social Communication & Environmental Interaction), Emotional Display (Facial, Verbal), and Interfering Behaviors (Aggression, Overactivity, Isolation). The OHAIRE-V3 assessment will be used to calculate a human-animal bond score to quantify the interactions taking place between the pediatric participants and the animal. The ELAN Coding software will be used to achieve high inter-rater reliability through precise timing of observed interactions. All AAT sessions will be recorded after receiving consent from families to retroactively code participant behavior during sessions.

- **How long are the procedures likely to take? Include duration and frequency.**

The A4A weekly therapy sessions will last about 30 minutes; however only it is expected that the total HAI time will be approximately 10-15 minutes, depending on the therapy activity. Honors students will assist the canine with Dr. Kilmer during the sessions and will take the canine to Dr. Kilmer's office once the HAI part of the appointment has ended.

- **How will information be given to people to get their informed consent to participate in this research? Answers should include specific methods (e.g., verbal consent, information handout, online consent form, full consent form requiring signature documentation.) **Please note that consent materials -- from a script for verbal consent to full consent forms that require participant signature -- must be uploaded in the Notes and Attachments section.**

  Dr. Kilmer will contact parents/guardians of potential participants to explain the AAT program and gage their interest. If interested, parents will come to the A4A clinic on the first floor of ECHP to meet the canine and sign the informed consent form. Verbal assent will be obtained from pediatric patients who are cognitively able to give it.

- **Does data collection rely on a scheduled event, such as a convention or specific date?**

  No

- **How will your data be collected? Include all that apply: online, on paper/in person, audio and/or video recordings. **Please note that all data collection materials will need to be uploaded in the Notes and Attachments section. This includes: surveys, questionnaires, interview questions or anything that is given to or asked of a participant.**

  Data will be collected in the initial consultation form for the A4A clinic, through performing developmental assessments, and with surveys. All AAT sessions will be recorded after receiving consent from families to retroactively code participant behavior during sessions. The following will be used to collect data: 1. A4A Intake form: all participants 2. Ages and Stages Questionnaire- 3rd edition: 1 month to 5.5 years 3. Ages and Stages Questionnaire: Social/Emotional- 2nd edition: birth to age 6 years 4. Social and Emotional Assessment/ Measure: 2 to 66 months 5. Strengths and Difficulties Questionnaire: 3 to 16 years 6. Positive and Negative Affect Schedule: 9 to 14 years 7. Vineland- 3rd edition: 3 to 21 years 8. Social Responsiveness Scale- 2nd edition: 2.5 to 18 years 9. PEDS: DM-AL: 7 to 11 years 10. Standford-Binet IQ Test: 2 to 18 years

- **How will your data be stored? Include all that apply: electronically, on paper, audio and/or video recordings.**

  Survey data will be stored in REDCap, a HIPAA-secured software which caregivers of youth with complete on a personal device (iPad, laptop, cellphone). Data will only be accessible by approved research staff. Data obtained in the ASQ- 3, ASQ: SE-2, SEAM, Vineland- 3, Strengths and Difficulties Questionnaire, and the Positive and Negative Affect Schedule scores will be stored in Dr. Kilmer's password protected laptop. Any hard-copy measures of documents will be stored in Drs. Kilmer or Quetsch's locked lab space in a locked filing cabinet. Video files will be downloaded from camcorders directly to Dr. Quetsch's locked lab space located in Memorial Hall on the password-protected computers and then additionally secured as a password protected file. Only authorized research personnel will have access to these files.

- **How will that data be kept secure?**

  Data will be stored on Drs. Kilmer and Quetsch's password-protected computers. Any hard-copy measures of documents will be stored in Dr. Quetsch's locked lab space in a locked filing cabinet. Only participant numbered IDs will be included on assessment documents. Participant identifying information and associated ID numbers will be stored in the Quetsch lab in a password protected computer on a password protected document. Only Drs. Quetsch and Kilmer will have access to participant ID information while the remaining research staff will utilize participant ID numbers. All video files will be downloaded to Dr.

Quetsch's lab computers within 24 hours and then immediately deleted from the camcorder device. Video files will be stored on a password-protected computer as password-protected documents.

- **Minimal Risk is defined as risks of harm not greater than those ordinarily encountered in daily life or during the performance of routine physical or psychological examinations or tests. Will participants be exposed to more than minimal risk? Include in your consideration the potential of mental risks if asking sensitive questions, or legal or reputational risks in case of breach of confidentiality.**
  Yes

  - **Describe the risks in question and any precautions that will be taken to minimize those risks.**
    Participants will not be exposed to more than minimal risks while receiving developmental or behavioral evaluations. Participants will be exposed to the canine during therapy sessions for approximately 10 to 15 minutes weekly. The canine will be removed if signs of fatigue, fright, or irritation appear. He will remain on leash throughout the entire AAT session and will be wearing a harness. Honors students will have the canine's leash during the AAT session and will hold him by the harness handle. Also, Dr. Kilmer will assess the disposition of the pediatric participants before allowing the canine to come to the room and will not allow the canine to participant if the pediatric participant's behavior is disruptive or potentially dangerous for the canine.

- **Are there any direct benefits to the participants for participating in this study?**
  Yes

  - **Describe the benefits participants will or may receive.**
    Pediatric participants will receive one plush toy that looks like the canine they are working with.

- **Will the proposed research involve deception or the withholding of information from participants?**
  No

- **Will the proposed research necessitate medical clearance from a physician prior to participation?**
  No

- **Will the proposed research involve gathering biological samples (blood, tissue, etc.)?**
  No

- **Will the proposed research involve administering of substances or providing food and drink, other than water, to participants?**
  No

- **Will the proposed research involve physical exercise or conditioning?**
  No

- **Does the research require review by a non-UofA IRB?**
  No

- **Does this research require approval from another institution or agency, such as a school or privately owned business?**
  No

Protocol Number: 2201382953 **Expiration Date:**
Investigator: Michele R Kilmer **Last Approval Date:** 04/01/2023

New/Changed Attachments

Description	Last Updated	Updated By
C-BARQ Assessment	01/25/2022 17:24:54	michelek@uark.edu
PANAS	01/25/2022 17:27:14	michelek@uark.edu
Intake form	01/25/2022 17:46:31	michelek@uark.edu
Demographic data	02/10/2022 21:20:48	michelek@uark.edu
Informed Consent form	04/27/2022 10:03:30	iwindwal@uark.edu

Actions

Description	Comments	Action Date
Amendment Created	Amendment-004: Created	05/08/2023
Expedited Approval	Renewal-001: Approved	03/13/2023
Expedited Approval	Renewal-001:	03/13/2023
Assigned to Agenda	Renewal-001:	03/13/2023
Submitted to IRB	Renewal-001: Submitted to IRB	02/07/2023
Renewal Created	Renewal-001: Created	02/07/2023
Renewal Reminder Generated	Renewal Reminder Letter #1	02/03/2023
Expedited Approval	Amendment-003: Approved	01/27/2023
Expedited Approval	Amendment-003:	01/27/2023
Assigned to Agenda	Amendment-003:	01/27/2023
Administrative Correction	Amendment-003: Making edit for reviewer clarification.	01/20/2023
Submitted to IRB	Amendment-003: Submitted to IRB	01/19/2023
Amendment Created	Amendment-003: Created	01/19/2023
Expedited Approval	Amendment-002: Approved	10/05/2022

Protocol Number: 2201382953 **Expiration Date:**
Investigator: Michele R Kilmer **Last Approval Date:** 04/01/2023

Actions

Description	Comments	Action Date
Expedited Approval	Amendment-002:	10/05/2022
Assigned to Agenda	Amendment-002:	10/05/2022
Submitted to IRB	Amendment-002: Submitted to IRB	09/25/2022
Amendment Created	Amendment-002: Created	09/25/2022
Expedited Approval	Amendment-001: Approved	09/16/2022
Assigned to Agenda	Amendment-001:	09/16/2022
Expedited Approval	Amendment-001:	09/16/2022
Submitted to IRB	Amendment-001: Submitted to IRB	08/18/2022
Amendment Created	Amendment-001: Created	08/02/2022
Expedited Approval		04/27/2022
Assigned to Agenda		04/27/2022
Administrative Correction	Changing consent document format to PDF for approval watermark.	04/27/2022
Submitted to IRB	Submitted to IRB	04/11/2022
Specific Minor Revisions Required		04/10/2022
Submitted to IRB	Submitted to IRB	03/30/2022
Substantive Revisions Required		03/16/2022
Submitted to IRB	Submitted to IRB	02/11/2022
Protocol Created	Protocol created	01/25/2022

Review Comments

**Protocol Number:**	2201382953	**Sequence Number:**	6
**Principal Investigator:**	Michele R Kilmer		
**Title:**	The Effect of Animal-Assisted Therapy on Prosocial Behavior in Children with Developmental Delay or Autism Spectrum Disorder		
**Committee Id:**	200	**Committee Name:**	IRB Expedited Review
**Schedule Id:**	33574	**Schedule Date:**	04/15/2023

Review Comments:

Canine Behavioral Assessment & Research Questionnaire (short version)

SECTION 1: Excitability

INSTRUCTIONS: Some dogs show little reaction to exciting events, while others become highly excited at the slightest novelty. By circling a number on the following 5-point scales (0=Calm, 4=Extremely excitable), please indicate your own dog's recent tendency to become excitable in the following circumstances **(please circle only one number)**:

1. Just before being taken for a walk.

Mild—Moderate excitability

Calm: little or no special reaction 0..............1..............2..............3..............4 **Extremely excitable:** over-reacts, hard to calm down.

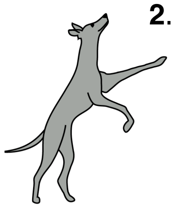

2. Just before being taken on a car trip.

Mild—Moderate excitability

Calm: little or no special reaction 0..............1..............2..............3..............4 **Extremely excitable:** over-reacts, hard to calm down.

SECTION 2: Aggression

INSTRUCTIONS: Most dogs display aggressive behavior from time to time—e.g. barking, growling, baring teeth, snapping, etc. By circling a number on the following 5-point scales (0= No aggression, 4= Serious aggression), please indicate your own dog's recent tendency to display aggressive behavior in each of the following circumstances **(please circle only one number)**:

3. When approached directly by an unfamiliar **person** while being walked/exercised on a leash.

Moderate aggression: growling/barking—baring teeth

No aggression: No visible signs of aggression 0..............1..............2..............3.............4 **Serious aggression:** Snaps, bites or attempts to bite.

4. When toys, bones or other objects are taken away by a household member.

Moderate aggression: growling/barking—baring teeth

No aggression: No visible signs of aggression 0..............1..............2..............3.............4 **Serious aggression:** Snaps, bites or attempts to bite.

5. When approached directly by a household member while s/he (the dog) is eating.

Moderate aggression: growling/barking—baring teeth

No aggression: No visible signs of aggression 0..............1..............2..............3.............4 **Serious aggression:** Snaps, bites or attempts to bite.

© James A. Serpell, University of Pennsylvania

6. When mailmen or other delivery workers approach your home.

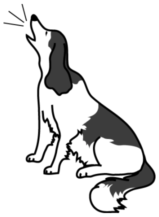

Moderate aggression:
growling/barking—baring teeth

No aggression: No visible signs of aggression 0..............1..............2..............3.............4 **Serious aggression:** Snaps, bites or attempts to bite.

7. When his/her food is taken away by a household member.

Moderate aggression:
growling/barking—baring teeth

No aggression: No visible signs of aggression 0..............1..............2..............3.............4 **Serious aggression:** Snaps, bites or attempts to bite.

8. When approached directly by an unfamiliar **dog** while being walked/exercised on a leash.

Moderate aggression:
growling/barking—baring teeth

No aggression: No visible signs of aggression 0..............1..............2..............3.............4 **Serious aggression:** Snaps, bites or attempts to bite.

9. When strangers walk past your home when your dog is outside or in the yard.

Moderate aggression:
growling/barking—baring teeth

No aggression: No visible signs of aggression 0..............1..............2..............3.............4 **Serious aggression:** Snaps, bites or attempts to bite.

10. When barked, growled, or lunged at by another (unfamiliar) dog.

Moderate aggression:
growling/barking—baring teeth

No aggression: No visible signs of aggression 0..............1..............2..............3.............4 **Serious aggression:** Snaps, bites or attempts to bite.

11. When approached while eating by another (familiar) household **dog** (leave blank if no other dogs).

Moderate aggression:
growling/barking—baring teeth

No aggression: No visible signs of aggression 0..............1..............2..............3.............4 **Serious aggression:** Snaps, bites or attempts to bite.

12. When approached while playing with/chewing a favorite toy, bone, object, etc., by another (familiar) household **dog** (leave blank if no other dogs).

Moderate aggression:
growling/barking—baring teeth

No aggression: No visible signs of aggression 0..............1..............2..............3.............4 **Serious aggression:** Snaps, bites or attempts to bite.

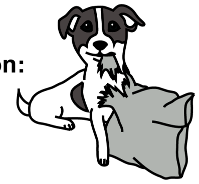

© James A. Serpell, University of Pennsylvania

SECTION 3: Fear and Anxiety

INSTRUCTIONS: Dogs often show signs of anxiety or fear when exposed to particular sounds, objects, persons or situations—e.g. crouching or cringing with tail tucked between the legs; whimpering or whining, freezing, trembling, or attempting to escape or hide. Using the following 5-point scales (0=No fear, 4=Extreme fear), please indicate your own dog's recent tendency to display fearful behavior in the following circumstances **(please circle only one number)**:

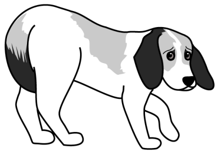

13. When approached directly by an unfamiliar person while away from your home.

No fear/anxiety: No visible signs of fear 0..............1..............2..............3..............4 **Extreme fear:** cowers; retreats or hides, etc.
Mild—Moderate fear/anxiety

14. In response to sudden or loud noises (e.g. thunder, vacuum cleaner, car backfire, road drills, objects being dropped, etc.).

No fear/anxiety: No visible signs of fear 0..............1..............2..............3..............4 **Extreme fear:** cowers; retreats or hides, etc.
Mild—Moderate fear/anxiety

15. When an unfamiliar person tries to touch or pet the dog.

No fear/anxiety: No visible signs of fear 0..............1..............2..............3..............4 **Extreme fear:** cowers; retreats or hides, etc.
Mild—Moderate fear/anxiety

16. In response to strange or unfamiliar objects on or near the sidewalk (e.g. plastic trash bags, leaves, litter, flags flapping, etc.

No fear/anxiety: No visible signs of fear 0..............1..............2..............3..............4 **Extreme fear:** cowers; retreats or hides, etc.
Mild—Moderate fear/anxiety

17. When approached directly by an unfamiliar dog.

No fear/anxiety: No visible signs of fear 0..............1..............2..............3..............4 **Extreme fear:** cowers; retreats or hides, etc.
Mild—Moderate fear/anxiety

18. When first exposed to unfamiliar situations (e.g. first car trip, first time in elevator, first visit to veterinarian, etc.).

No fear/anxiety: No visible signs of fear 0..............1..............2..............3..............4 **Extreme fear:** cowers; retreats or hides, etc.
Mild—Moderate fear/anxiety

19. When barked, growled, or lunged at by an unfamiliar dog.

No fear/anxiety: No visible signs of fear 0..............1..............2..............3..............4 **Extreme fear:** cowers; retreats or hides, etc.
Mild—Moderate fear/anxiety

20. When having nails clipped by a household member.

No fear/anxiety: No visible signs of fear	Mild—Moderate fear/anxiety	Extreme fear: cowers; retreats or hides, etc.
	0..............1..............2..............3..............4	

21. When groomed or bathed by a household member.

No fear/anxiety: No visible signs of fear	Mild—Moderate fear/anxiety	Extreme fear: cowers; retreats or hides, etc.
	0..............1..............2..............3..............4	

SECTION 4: Separation-related behavior.

INSTRUCTIONS: Some dogs show signs of anxiety when left alone, even for short periods of time. Thinking back over the recent past, how often has your dog shown each of the following signs of anxiety when left, or about to be left, on its own **(please check only one box per question)**:

	Never	Seldom	Sometimes	Usually	Always
**22.** Restlessness/agitation/pacing.	☐	☐	☐	☐	☐
**23.** Barking or whining.	☐	☐	☐	☐	☐
**24.** Chewing/scratching at doors, floor, windows, curtains, etc.	☐	☐	☐	☐	☐

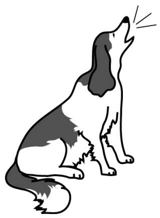

SECTION 5: Attachment and Attention-seeking.

INSTRUCTIONS: Most dogs are strongly attached to their people, and some demand a great deal of attention and affection from them. Thinking back over the recent past, how often has your dog shown each of the following signs of attachment or attention-seeking **(please check only one box per question)**:

	Never	Seldom	Sometimes	Usually	Always
**25.** Tends to follow you (or other members of the household) about the house, from room to room.	☐	☐	☐	☐	☐
**26.** Tends to sit close to, or in contact with, you (or others) when you are sitting down	☐	☐	☐	☐	☐

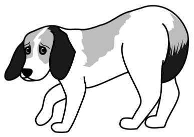

SECTION 6: Training and obedience

INSTRUCTIONS: Some dogs are more obedient and trainable than others. By checking the appropriate boxes, please indicate how trainable or obedient your dog has been in each of the following situations in the recent past **(please check only one box per question)**:

	Never	Seldom	Sometimes	Usually	Always
**27.** Obeys a "sit" command immediately.	☐	☐	☐	☐	☐
**28.** Obeys a "stay" command immediately.	☐	☐	☐	☐	☐
**29.** Easily distracted by interesting sights, sounds or smells.	☐	☐	☐	☐	☐

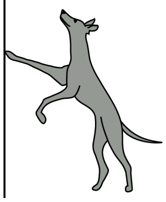

SECTION 7: Miscellaneous problems

INSTRUCTIONS: Dogs display a wide range of miscellaneous behavior problems in addition to those already covered by this questionnaire. Thinking back over the recent past, please indicate how often your dog has shown any of the following behaviors **(please check only one box per question)**:

	Never	Seldom	Sometimes	Usually	Always
**30.** Chases or would chase birds, given the chance.	☐	☐	☐	☐	☐
**31.** Chases or would chase squirrels, rabbits, etc., given the chance.	☐	☐	☐	☐	☐
**32.** Escapes or would escape from home or yard, given the chance.	☐	☐	☐	☐	☐
**33.** Chews inappropriate objects.	☐	☐	☐	☐	☐
**34.** Pulls excessively hard when on the leash.	☐	☐	☐	☐	☐
**35.** Urinates against objects/ furnishings in your home.	☐	☐	☐	☐	☐
**36**. Urinates when left alone at night, or during the daytime.	☐	☐	☐	☐	☐
**37**. Defecates when left alone at night, or during the daytime.	☐	☐	☐	☐	☐

© James A. Serpell, University of Pennsylvania

**38.** Hyperactive, restless, has trouble settling down.	☐	☐	☐	☐	☐
**39.** Playful, puppyish, boisterous.	☐	☐	☐	☐	☐
**40.** Active, energetic, always on the go.	☐	☐	☐	☐	☐
**41.** Chases own tail/hind end.	☐	☐	☐	☐	☐
**42**. Barks persistently when alarmed or excited.	☐	☐	☐	☐	☐

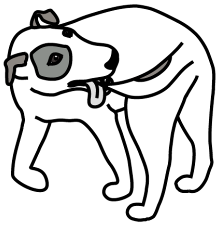

Thank you for providing this helpful information!

© James A. Serpell, University of Pennsylvania

THE OHIO STATE UNIVERSITY

Positive and Negative Affect Schedule (PANAS-SF)

Indicate the extent you have felt this way over the past week.		Very slightly or not at all	A little	Moderately	Quite a bit	Extremely
PANAS 1	Interested	☐ 1	☐ 2	☐ 3	☐ 4	☐ 5
PANAS 2	Distressed	☐ 1	☐ 2	☐ 3	☐ 4	☐ 5
PANAS 3	Excited	☐ 1	☐ 2	☐ 3	☐ 4	☐ 5
PANAS 4	Upset	☐ 1	☐ 2	☐ 3	☐ 4	☐ 5
PANAS 5	Strong	☐ 1	☐ 2	☐ 3	☐ 4	☐ 5
PANAS 6	Guilty	☐ 1	☐ 2	☐ 3	☐ 4	☐ 5
PANAS 7	Scared	☐ 1	☐ 2	☐ 3	☐ 4	☐ 5
PANAS 8	Hostile	☐ 1	☐ 2	☐ 3	☐ 4	☐ 5
PANAS 9	Enthusiastic	☐ 1	☐ 2	☐ 3	☐ 4	☐ 5
PANAS 10	Proud	☐ 1	☐ 2	☐ 3	☐ 4	☐ 5
PANAS 11	Irritable	☐ 1	☐ 2	☐ 3	☐ 4	☐ 5
PANAS 12	Alert	☐ 1	☐ 2	☐ 3	☐ 4	☐ 5
PANAS 13	Ashamed	☐ 1	☐ 2	☐ 3	☐ 4	☐ 5
PANAS 14	Inspired	☐ 1	☐ 2	☐ 3	☐ 4	☐ 5
PANAS 15	Nervous	☐ 1	☐ 2	☐ 3	☐ 4	☐ 5
PANAS 16	Determined	☐ 1	☐ 2	☐ 3	☐ 4	☐ 5
PANAS 17	Attentive	☐ 1	☐ 2	☐ 3	☐ 4	☐ 5
PANAS 18	Jittery	☐ 1	☐ 2	☐ 3	☐ 4	☐ 5
PANAS 19	Active	☐ 1	☐ 2	☐ 3	☐ 4	☐ 5
PANAS 20	Afraid	☐ 1	☐ 2	☐ 3	☐ 4	☐ 5

Scoring:

Positive Affect Score: Add the scores on items 1, 3, 5, 9, 10, 12, 14, 16, 17, and 19. Scores can range from 10 – 50, with higher scores representing higher levels of positive affect.
Mean Scores: 33.3 (SD±*7.2)*

Negative Affect Score: Add the scores on items 2, 4, 6, 7, 8, 11, 13, 15, 18, and 20. Scores can range from 10 – 50, with lower scores representing lower levels of negative affect.
Mean Score: 17.4 (SD ± 6.2)

Your scores on the PANAS: Positive: ____ Negative: ____

Watson, D., Clark, L. A., & Tellegen, A. (1988). Development and validation of brief measures of positive and negative affect: the PANAS scales. *Journal of personality and social psychology*, *54*(6), 1063.

CO-INVESTIGATORS

Michele Kilmer, Assistant Professor
The University of Arkansas, Eleanor Mann School of Nursing
606 N. Razorback Rd.
1-479-575-3904
michelek@uark.edu

Lauren Quetsch, Assistant Professor
The University of Arkansas, Fulbright College of Arts and Sciences
Department of Psychological Science
216 Memorial Hall,
1-479-575-5817
quetsch@uark.edu

PURPOSE OF STUDY

This purpose of this study is to evaluate the effect of services for children with developmental delay or autism spectrum disorder while using a dog trained in therapeutic techniques. This study's aim is to see if therapy sessions with the dog result in an improvement in your child's social and emotional abilities. Before you decide to allow your child to participate in this study, it is important that you understand why the study is being done and what will be involved. Please read the following information carefully. Please ask the co-investigator if there is anything that is not clear or if you need more information.

DESCRIPTION OF STUDY

Activities involved in this study include weekly therapy sessions lasting 30 minutes that will assist your child with their identified social and emotional concerns. Your child will receive 24 weeks of therapy, where 12 weeks will be with the dog and 12 weeks will be without the dog. You will be asked to complete a set of assessments on your child's development, social-emotional skills, functioning, parent-child interactions, and intelligence quotient throughout the study. If able, your child will indicate their level of anxiety before and after each therapy session using a picture of a thermometer with varying levels of anxiety. Adolescents may be asked to self-report how they feel their socialization and emotional behaviors were during the previous week.

Animal-assisted therapy sessions include techniques aimed to help your child with identified concerns in social and emotional behavior. The dog is trained to assist your child to engage in social interaction, like playing gently and taking turns during a game. Animal-assisted activities include playing fetch and other turn-taking games, assisting the dog to complete puzzles, coloring pictures with the dog, petting the dog, and grooming the dog. The dog can also give "hugs" if your child is anxious by leaning his body gently against your child's legs. Free play with the dog will be allowed once your child and dog have built a relationship. The sessions with the dog will last between 10 and 15 minutes, then the remaining time will be spent discussing home therapy techniques to practice until the next session. Research personnel who have been training the dog may be present to assist the dog during the session, and Dr. Kilmer, the dog's owner and handler, will be present while the dog is in the therapy session with your child.

Traditional therapy sessions without the dog will last for 30 minutes and will focus on home therapeutic techniques to identify and improve social and emotional concerns. All therapy sessions in the clinic will be video-recorded for research purposes.

RISKS

Minimal risks are associated with these studies. Other than the normal emotional risks associated with therapy of this type, the only risk involved with this research is normal minimal risk involved with interacting with a well-behaved dog The dog is being trained as a therapy and service dog and attends weekly sessions with experienced trainers. Dr. Kilmer is being educated to properly perform animal-assisted therapy sessions. The dog will be on leash throughout the session and research personnel will be present to remove the dog if he or your child appear to be distressed. The research personnel have been involved in the dog’s training and will be constantly assessing his behavior and can remove him as needed. There is a very small risk of injury from the dog (e.g., scratching, teething/nipping); however, given the extensive training of the dog and continuous monitoring of the dog at all times, this risk is considered minimal and unlikely.

BENEFITS

There are no direct benefits to you for allowing your child's assessments to be used in this research; however, results from this research could improve therapeutic care of children with developmental delay or autism spectrum disorder. Your child will also receive a plush toy that looks like the dog for participating in the study.

CONFIDENTIALITY

Participant data will be kept confidential to the extent allowed by law and University policy. Your responses to the questionnaires and evaluations will have all identifying information removed. The co-investigators will keep data in their computers that are password protected. Notes, interview transcriptions, and any other hard-copies of identifying participant information will be secured in a locked file cabinet in the personal possession of the co-investigators. Videos of therapeutic sessions will be downloaded to co-investigators’ password-protected university-issued computers within 24 hours and then immediately deleted from the camcorder device. Only research personnel will have access to these files, unless otherwise required by law. Co-investigators are legally obligated to report specific incidents which include, but may not be limited to, incidents of abuse and suicide risk.

Your child has been identified at-risk for developmental delay or autism spectrum disorder by his/her primary care provider (PCP), who then referred your child to the EMSON Pediatric Health Lab for further developmental assessment. As such, the referring PCP will receive the results of the assessments as well as recommendations for identified concerns. Also, the investigators may request your consent to obtain medical records from the referring provider, allied health, and specialty clinics who take care of your child. Your child’s school teacher and staff may be contacted to provide records or complete forms necessary for the evaluations if indicated. Aside from this, the co-investigators request permission to use assessment results in research on pediatric autism and developmental delay.

CONTACT INFORMATION

If you have questions at any time about this study, or you experience adverse effects as the result of participating in this study, you may contact the co-investigators, whose contact information is provided on the first page. If you have questions regarding your rights as a study participant, or if problems arise which you do not feel you can discuss with the co-investigators, please contact the University of Arkansas Institutional Review Board at 1-479-575-2208.

VOLUNTARY PARTICIPATION

Both your and your child's participation in this study is voluntary. It is your decision whether or not to allow your child to take part in this study. If you decide to allow your child to take part in this study, you will be asked to sign this consent form. If your child is able, they will also be asked to provide their assent. After you sign this consent form, you or your child are still free to stop your participation or to leave at any time and without giving a reason. Withdrawing from this study will not affect the relationship you have, if any, with the co-investigators.

CONSENT

I have read and I understand the provided information and have had the opportunity to ask questions. I understand that my participation is voluntary and that I am free to withdraw at any time, without giving a reason and without cost. I understand that I will be given a copy of this consent form. I voluntarily agree to take part in this study.

Parent/Guardian signature ___________________________________ Date ___________

Child assent:

I have discussed this study with my parent/guardian, and I agree to participate. I understand that even if they agree, it's okay if I choose not to participate or change my mind about participating later.

Child signature ____________________________________ Date ____________

Investigator signature _______________________________ Date _____________
